# In Vitro Screening of an FDA‐Approved Chemical Library for Arylsulfatase A Using Fibroblast Cells Derived From Patients With MLD

**DOI:** 10.1155/bmri/1945300

**Published:** 2026-10-07

**Authors:** Takashi Miyajima, Miki Igarashi, Chen Wu, Takeo Iwamoto, Yoshikatsu Eto

**Affiliations:** ^1^ Advanced Clinical Research Center Southern TOHOKU Research Institute for Neuroscience Kawasaki Japan minamitohoku.or.jp; ^2^ Institute of Medical Science Tokyo Medical University Tokyo Japan tokyo-med.ac.jp; ^3^ The Jikei University School of Medicine Tokyo Japan jikei.ac.jp

**Keywords:** arylsulfatase A, drug screening, metachromatic leukodystrophy

## Abstract

**Background:**

Metachromatic leukodystrophy (MLD) is a rare metabolic disease characterized by demyelination and inherited in an autosomal recessive manner. It is caused by mutations in the arylsulfatase A (*ARSA*) gene. When ARSA enzyme activity is lacking or reduced, sulfatides and lysosulfatides accumulate, particularly within the myelin sheath, resulting in various symptoms, including developmental delay and movement disorders. Gene therapy using hematopoietic stem cells has been developed and approved for MLD; however, the long‐term risks associated with lentiviral vectors remain uncertain. Therefore, expanding the available therapeutic options for patients with MLD remains essential.

**Methods:**

This study is aimed at exploring two potential drugs that may induce heat‐shock protein, along with 765 FDA‐approved drugs that might enhance ARSA enzyme activity, using four fibroblast cell lines derived from patients with MLD.

**Results:**

Fibroblasts derived from patients with MLD exhibited low ARSA enzyme activities and increased sulfatide accumulation. Geranylgeranylacetone, an HSP70 inducer, increased the enzyme activity, while arimoclomol, an HSP coinducer, did not affect it. Furthermore, a screening test revealed that two of the 765 FDA‐approved drugs increased the enzyme activity in MLD fibroblasts.

**Conclusion:**

These results lay the groundwork for future research into the development of new therapeutic options for MLD.

## 1. Introduction

Metachromatic leukodystrophy (MLD; OMIM #250100) is a metabolic disorder inherited in an autosomal recessive pattern. It is characterized by demyelination and is caused by mutations in the arylsulfatase A (*ARSA*) gene. Reduced ARSA enzyme activity leads to sulfatides and lysosulfatides accumulation in various tissues, particularly within the myelin sheath. Demyelination occurs in either central or peripheral nerves, causing wide‐ranging symptoms, including developmental delays, movement disorders, spastic paralysis, and seizures [1–3]. MLD is classified into late‐infantile, juvenile, and adult forms based on age at onset. The late‐infantile form, the most common subtype, typically presents at 18–24 months with gait disturbance, followed by rapid deterioration of motor, speech, and cognitive function, with death usually occurring within 5–6 years of onset. Once early‐onset patients become symptomatic, no available therapy effectively halts disease progression, making early intervention critical. Nonetheless, several approaches, including gene therapy, are under development. Hematopoietic stem cell transplantation for presymptomatic or early‐stage MLD can ameliorate white matter damage, delay disease onset, and slow disease progression [4, 5]. Although preliminary safety and tolerability results of intrathecal enzyme replacement therapy have been reported, the treatment′s effectiveness in halting disease progression remains unclear [6]. Additionally, atidarsagene autotemcel, consisting of autologous CD34+ hematopoietic stem and progenitor cells transduced ex vivo with a lentiviral vector encoding ARSA cDNA, has shown promising outcomes [7]. However, these approaches carry substantial transplantation‐related risks and costs, and their benefit is largely limited to presymptomatic or very early‐stage patients, underscoring the need for therapies that can be initiated even earlier in the disease course.

Moreover, lysosomal storage diseases may be treated with drugs using small molecules that function as pharmacological chaperones, substrate synthesis inhibitors, and autophagy and proteostasis regulators. Drugs such as migalastat (Galafold) for Fabry disease, miglustat (Brazaves) and eliglustat (Cerdelga) for Gaucher disease, and miglustat, eliglustat, and arimoclomol for Niemann–Pick disease type C (NPC) have been developed and approved [8–11]. Having multiple treatment options enhances patient choice and improves their quality of life (QOL). To expand treatment options for MLD, this study is aimed to evaluate the effects of two proteostasis drugs, namely, arimoclomol and geranylgeranylacetone (GGA), on the ARSA enzyme activity in skin fibroblasts obtained from patients with MLD and screen potential molecules in 765 FDA‐approved drugs for MLD therapy.

## 2. Material and Methods

### 2.1. Fibroblast Culture

We obtained fibroblasts from healthy subjects (GM07525 and GM22159) and patients with MLD (GM00197, GM00243, GM00905, and GM10199) through the Coriell Cell Repository (Camden, New Jersey, United States) (Table 1). These fibroblasts were cultured in Dulbecco′s Modified Eagle′s Medium (Merck Millipore, Massachusetts, United States) supplemented with 10% fetal bovine serum (Thermo Fisher Scientific, Massachusetts, United States) and penicillin‐streptomycin solution (1×; FUJIFILM Wako Pure Chemical Corporation, Osaka, Japan) at 37°C with 95% humidity and 5% CO_2_.

**Table 1:** Basic information of fibroblasts.

Fibroblast	Sex	Race	Age at sampling	Disease	Mutation	References	Protein	Phenotype	ARSA enzyme activity nmol/mg protein/h
GM07525	Female	White	22 y	—	—			—	186.1
GM22159	Male	Asian	1 day	—	—			—	235.9
GM00197	Male	White	4 y	MLD	c.684+1G>A c.1283C>T	[12] [13]	spl. p.P428L	Infantile type	14.3
GM00243	Male	White	3 y	MLD	c.465+1G>A c.465+1G>A	[14]	spl. spl.	Infantile type	9.8
GM00905	Female	White	3 y	MLD	c.465+1G>A c.465+1G>A	[14]	spl. spl.	Infantile type	7.7
GM10199	Female	White	11 y	MLD	c.499C>T c.577C>A	[15]	p.P150L p.P193T	Late onset than juvenile type	22.3

Abbreviation: ARSA, arylsulfatase A.

### 2.2. Mutation Analysis

Genomic DNA was extracted from the fibroblasts using the DNeasy Blood and Tissue Kit (Qiagen N.V., Venlo, Netherlands). The Platinum Taq DNA Polymerase High Fidelity (Thermo Fisher Scientific) and the Wizard SV Gel and Clean‐Up System (Promega, Wisconsin, United States) were used for amplifying all exons of the *ARSA* gene and purifying the resulting products of polymerase chain reaction (PCR), respectively. Sequencing services were outsourced to Eurofins Genomics Inc. (Tokyo, Japan).

To amplify the full cDNA length of *ARSA* gene, we extracted total RNA from fibroblasts using the RNA isoPlus (Takara Bio Inc., Shiga, Japan) and further purified it using the RNeasy Mini kit (Qiagen) with DNase. First‐strand cDNA was synthesized from RNA by reverse transcription using the ReverTra Ace q‐PCR RT Master Mix (TOYOBO, Tokyo, Japan). Platinum Taq DNA Polymerase High Fidelity (Thermo Fisher Scientific) was employed for PCR. Samples were loaded into an agarose gel for electrophoresis to check the PCR product size. Primer sequences used for mutation analysis are listed in Table 2.

**Table 2:** Primer sequences.

	Forward	Reverse	Product
**Gene sequence**			
Exon 1–2	GTATTTGGGTCCGGGGTCTC	GTCACAGCCACCGTCGCAA	897 bp
Exon 3–4	GATTTCTAGGCATCCCGTACTC	CCCTCACCCACTATGTTCTTG	706 bp
Exon 5–6	GCCAAGAACATAGTGGGTGAGG	GGTAGAAGAAGAGAGACTGCCGAG	861 bp
Exon 7–8	GCAAGAAGCGGTGCACGTCC	TTCCTCATTCGTACCACAGGGGAC	831 bp
Exon 1–2 reverse	Sequence primer	GGGAAGCAGGTCAGGTTCTG	
**Q-PCR**			
*ARSA*	GCTCTACCACTCCCAACCTG	GCCATTCCTGTGAGGTAGCC	235 bp
*ACTB*	CATGTACGTTGCTATCCAGGC	CTCCTTAATGTCACGCACGAT	250 bp
**cDNA length**			
*ARSA*	ATGTCCATGGGGGCACCGCGGT	TCAGGCATGGGGATCTGGGCAAT	1530 bp

Abbreviation: ARSA, arylsulfatase A.

### 2.3. Measurement of ARSA Enzyme Activity and mRNA Expression

To assess ARSA enzyme activity, we homogenized fibroblasts in water by sonication and incubated the homogenates in 0.5 M acetate buffer (pH 5.0) containing 10 mM 4‐nitrocatechol sulfate (4‐NCS), 0.5 mM sodium pyrophosphate, and 10% NaCl for 1 h at 37°C. Next, we added 1 N NaOH to stop the reaction and measured the absorbance at 515 nm using a microplate reader (ARVO X3; PerkinElmer, Massachusetts, United States) [16]. Enzyme activity was normalized to protein concentration, which was determined using the Pierce BCA Protein Assay Kit (Thermo Fisher Scientific). To measure mRNA expression, we performed qPCR using TB Green Premix Ex Taq II (Takara Bio Inc., Shiga, Japan) with an MX3000P real‐time qPCR system (Stratagene, La Jolla, California, United States). The data were analyzed using the comparative cycle threshold method, and the mRNA levels of the target genes were normalized using the mRNA expression of ACTB as an internal control.

### 2.4. Western Blotting

ARSA protein expression was analyzed by Western blotting as described previously [17, 18]. Briefly, cells were lysed in the M‐PER Mammalian Protein Extraction Reagent (Thermo Fisher Scientific) supplemented with cOmplete Mini Protease Inhibitor Cocktail (Merck Millipore) and PhosSTOP (Merck Millipore) on ice for 15 min. Samples were centrifuged at 13,000 g for 15 min at 4°C. We used the supernatants for Western blotting and quantified protein concentrations using the Pierce BCA Protein Assay Kit. An aliquot of the supernatants was then mixed with SDS sample buffer and heated at 95°C for 5 min. The samples were loaded on SDS polyacrylamide gel (Mini‐PROTEAN TGX Precast Gel: Bio‐Rad, Hercules, California, United States). Subsequently, proteins were electrophoretically transferred to a polyvinylidene difluoride (PVDF) membrane (Trans‐Blot Turbo Mini PVDF Transfer Pack: Bio‐Rad). The blots were exposed to a blocking buffer (3% [w/v] nonfat dry milk in TBST) before its incubation with the following primary antibodies: ARSA antibody (ARP54528_P050, Aviva Systems Biology Corporation, San Diego, California, United States) and *β*‐actin antibody (#4967, Cell Signaling Technology, Inc., Danvers, Massachusetts, United States). These blots were further incubated with anti‐rabbit IgG, HRP‐linked secondary antibody (#7074 Cell Signaling Technology, Inc.). Protein bands were detected using ECL Select Western blotting detection reagent (Cytiva: GE Healthcare, Amersham, United Kingdom), and the luminescence was captured using an ImageQuant LAS 500 system (Cytiva).

### 2.5. Quantification of N‐C16:0‐Sulfatide

Cells were homogenized in 1 mL of phosphate‐buffered saline (PBS) using a sonicator. Protein content in the homogenized samples was measured using the BCA Protein Assay. Six volumes of 2‐propanol were added to the homogenized samples. The mixture was then vortexed for 10 min and centrifuged at 15,000 × g for 10 min at room temperature. The supernatant was mixed with internal standard (IS) solution (13C‐d7, 1.77 ng/*μ*L) and dried using a vacuum concentrator (SpeedVac). The residue was reconstituted in MeOH for analysis by ultrahigh performance liquid chromatography tandem mass spectrometry (UHPLC‐MS/MS).

Moreover, N‐C16:0‐sulfatide was quantified using ultraperformance liquid chromatography–tandem mass spectrometry (UPLC–MS/MS; H‐Class, Xevo TQ‐S Micro, Waters, United States). A C18 column (ACQUITY UPLC BEH C18, 1.7 *μ*m, 2.1 × 50 mm, Waters, United States) connected to an in‐line filter unit (ACQUITY, Waters, United States) was utilized for separation using an acetonitrile gradient system. Mobile Phases A and B were 10 mM ammonium formate solution and acetonitrile, respectively. The gradient was applied as follows: 0–2 min, 70%–100% linear gradient; 2–12 min, 100% acetonitrile; and 12–13 min, 100%–70% linear gradient. The flow rate was 0.2 mL/min. Column temperature was maintained at 40°C. We selected electrospray ionization negative ion source with source temperature at 150°C and desolvation temperature at 600°C. The multiple reaction‐monitoring transitions were 778.36 > 96.97 m/z for N‐C16:0‐sulfatide and 759.50 > 96.97 m/z for 13C‐d7. To quantify N‐C16:0‐sulfatide, we compared the peak molecular target area with the IS area.

### 2.6. Drug Effects

We tested two heat‐shock protein inducers, namely, arimoclomol (Sigma‐Aldrich) and GGA (Tokyo Chemical Industry Co. Ltd), on the ARSA enzyme activity of skin fibroblasts from patients with MLD. We also screened potential molecules in the FDA‐approved drug library V2 containing 765 compounds provided from AMED‐BINDS (Tokyo, Japan) for MLD therapy. The experiments utilized GM10199 cells derived from a patient with a late‐onset phenotype, who exhibited milder clinical symptoms than the infantile type. ARSA enzyme activity in this cell line was slightly higher than that in the other tested lines (Table 1). For testing either arimoclomol or GGA, GM10199 cells (Passages 13–15) were seeded in 48‐well plates at 4 × 10^4^/well and incubated for 24 h. Then, the cells were exposed to each drug at 50 or 200 *μ*M for 48 h. For drug screening, GM10199 cells (Passage 13) were seeded in 96‐well plates at 2 × 10^4^/well and incubated for 24 h. Subsequently, the cells were exposed to a drug at 5 *μ*M for 48 h. After incubation, these cells were washed with PBS and lysed in 0.1% Igepal CA‐630 (MP Biomedicals, Ohio, United States) (a lysis buffer) by shaking for 20 min. We then incubated the lysate in 0.5 M acetate buffer (pH 5.0) containing 10 mM 4‐NCS, 0.5 mM sodium pyrophosphate, and 10% NaCl for 1 or 16 h (drug screening) at 37°C [19]. Enzyme activity was calculated as described above. For drug screening, relative enzyme activity was calculated based on absorbance at 515 nm, and normalized to the dimethyl sulfoxide (DMSO)‐treated control wells.

### 2.7. Statistical Analysis

Measured values are expressed as means and standard errors of the means. All statistical data were analyzed using GraphPad Prism Version 7.04 (GraphPad Software Inc., La Jolla, California, United States). Statistical significance was assessed using unpaired Student′s *t*‐tests, Aspin–Welch test, and one‐way analysis of variance, as appropriate. Post hoc analyses were conducted using Dunnett′s multiple comparison test when significant differences were detected. A *p* value below 0.05 was considered statistically significant.

## 3. Results

First, we analyzed the *ARSA* gene mutations in the fibroblasts (Table 1). GM00197 possessed the missense variant c.1283C>T and the splicing variant c.684+1G>A. Both GM00243 and GM00905 exhibited the homozygous splicing variant c.465+1G>A. Meanwhile, GM10199 contained the missense variants c.499C>T and c.577C>A. Next, cDNA length was measured in individual fibroblasts. The full length of ARSA cDNA is 1530 base pairs (NCBI Reference Sequence: NM_000487.6), and a band of this size was confirmed in GM22159 and GM07525 derived from healthy subjects (Figure 1). In GM10199, which harbored missense mutations on both alleles, a single PCR band of 1530 bp was detected. GM00197 carried one missense mutation and one splicing mutation, producing one band at 1530 bp, along with additional shorter, aberrant transcripts. In GM00243 and GM00197, one aberrant and longer transcript was detected, whereas the other band appeared at approximately 1500 bp. Conversely, we did not check the sequence of the aberrant bands in GM00243, GM00905, and GM00197 deriving from splicing variants. Therefore, the protein and mRNA expression levels of *ARSA* were analyzed in the fibroblasts. All observed fibroblasts contained only one protein band, which was detected at approximately 55 kDa (Figure 2A). Protein expression levels were significantly increased in MLD fibroblasts such as GM00197, GM00243, and GM10199, but not GM00905, compared with those in GM22159 among healthy subjects (Figure 2A,B). The mRNA expression levels of *ARSA* were significantly lower in GM00243 and GM00905 than in GM22159 (Figure 2C). The expression levels were increased in GM10199 compared with those in GM22159; meanwhile, GM00197 showed no change. Finally, we measured ARSA enzyme activities in fibroblasts derived from both healthy subjects and patients with MLD (Table 1). Notably, ARSA enzyme activities were approximately 10 times lower in fibroblasts from patients with MLD than from healthy subjects. Additionally, the ARSA enzyme activity of GM10199, established in patients with late‐onset type MLD, was higher than those of GM00197, GM00243, and GM00905 derived from patients with infantile‐type MLD. This study also confirmed that ARSA enzyme activity in leukocytes was approximately three times lower in patients with MLD, who appeared symptomatic in infantile stage, than in carriers (data not shown). In addition, sulfatides accumulated by over four times in MLD fibroblasts compared with those in healthy subjects fibroblasts (Figure 3).

**Figure 1:**
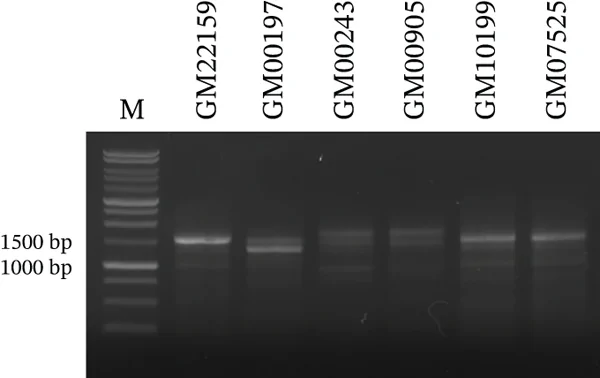
The *ARSA* coding region was amplified by PCR using cDNA templates derived from six fibroblast lines. The amplified coding region spans 1530 base pairs.

**Figure 2:**
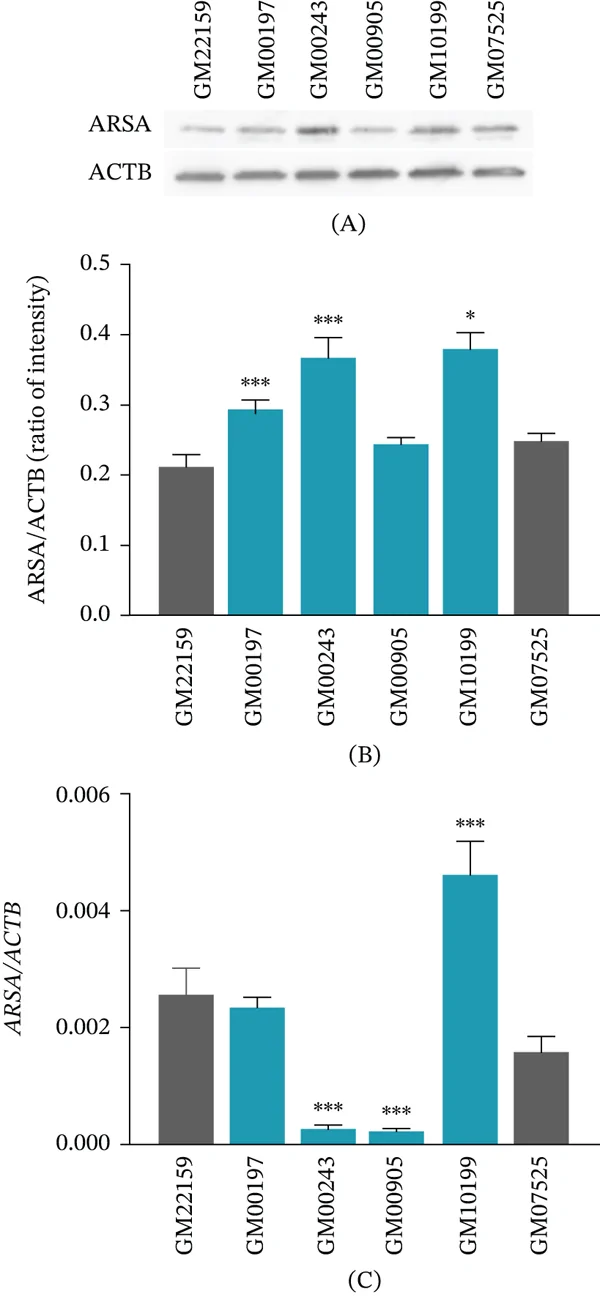
Alteration of the protein and mRNA expression levels of *ARSA* in metachromatic leukodystrophy (MLD)–derived fibroblasts. Protein and mRNA expression levels of ARSA in fibroblasts were analyzed by Western blotting and qPCR, respectively. (A) Representative immunoblots, (B) relative protein expression levels of ARSA, and (C) relative mRNA expression levels of *ARSA* in fibroblasts derived from healthy subjects and patients with MLD. Data are mean ± SEM. ∗*p* < 0.05 and ∗∗∗*p* < 0.001 compared with GM22159 (one‐way ANOVA followed by Dunnett′s test). *n* = 3 for (B) and *n* = 4 for (C).

**Figure 3:**
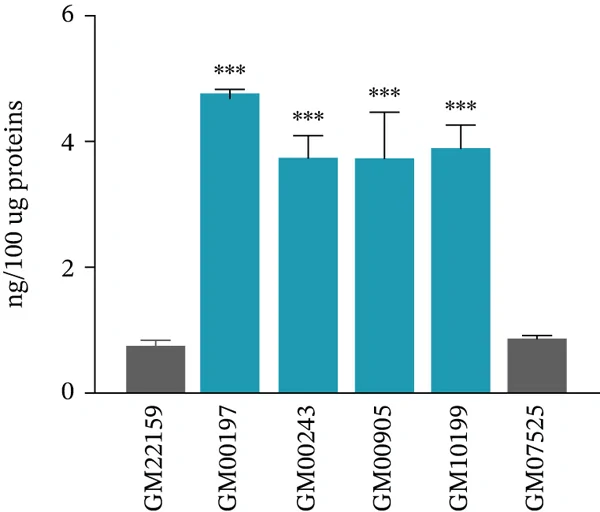
Accumulation of N‐hexadecanoyl‐sulfatide in MLD‐derived fibroblasts. Cellular N‐hexadecanoyl‐sulfatide was analyzed via liquid chromatography tandem mass spectrometry. Data are expressed as mean ± SEM (*n* = 3). ∗∗∗*p* < 0.001 compared with GM22159 (one‐way ANOVA followed by Dunnett′s test).

Next, we examined whether the heat‐shock protein inducers (GGA and arimoclomol) can modify ARSA enzyme activity in MLD‐derived fibroblasts. GGA treatment increased ARSA enzyme activity in GM10199, but not other MLD fibroblasts, by 1.23‐fold at 200 *μ*M concentration compared with that in DMSO‐treated control (Table 3). However, arimoclomol treatment did not modify the ARSA enzyme activity in all MLD‐derived fibroblasts (Table 4). GGA treatment reduced total ARSA protein levels in GM10199 fibroblasts, suggesting that GGA may promote clearance of misfolded/aggregated mutant ARSA protein (Figure 4), although direct evidence of reduced aggregation (e.g., insoluble fraction analysis) would be needed to confirm this interpretation.

**Table 3:** Effects of GGA on ARSA of MLD‐derived fibroblasts.

	Control	GGA (50 *μ*M)	GGA (200 *μ*M)
	(nmol/mg protein/h)		
GM22159	121.9 ± 3.2	120 ± 7.1	104.2 ± 5.1
GM00197	12.5 ± 0.4	—	12.6 ± 0.2
GM00243	11.8 ± 0.9	—	11.6 ± 1.1
GM00905	11.0 ± 2.9	10.4 ± 0.5	10.5 ± 0.4
GM10199	14.0 ± 0.9	14.1 ± 0.7	17.4 ± 0.3∗
GM07525	121.1 ± 7.7	—	93.6 ± 8.0

*Note:* Mean ± SEM (*n* = 3).

Abbreviations: ARSA, arylsulfatase A; GGA, geranylgeranylacetone; MLD, metachromatic leukodystrophy.

^∗^ *p* < 0.05 compared with the 0 μM control.

**Table 4:** Effects of arimoclomol on ARSA of fibroblasts derived from patients with MLD.

	Control	Arimoclomol
	(nmol/mg protein/h)	
GM22159	110.5 ± 12.1	111.1 ± 8.1
GM00197	13.9 ± 1.0	14.4 ± 0.4
GM00243	12.5 ± 1.1	11.0 ± 1.2
GM00905	8.4 ± 0.3	9.0 ± 0.1
GM10199	20.5 ± 1.6	17.9 ± 0.9
GM07525	124.8 ± 16.6	104.0 ± 2.1

*Note:* Mean ± SEM (*n* = 3). Arimoclomol, 200 *μ*M.

Abbreviations: ARSA, arylsulfatase A; MLD, metachromatic leukodystrophy.

**Figure 4:**
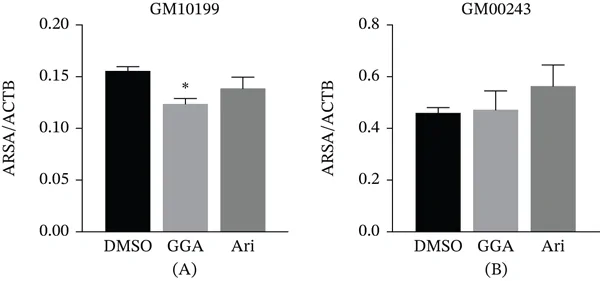
Protein levels in the fibroblasts treated with heat‐shock protein modulators. Cells were treated with either geranylgeranylacetone or arimoclomol, and ARSA protein levels were analyzed in the cells by Western blotting. Relative expression of ARSA in (A) GM10199 and (B) GM00243. Data are expressed as mean ± SEM (*n* = 3). ∗*p* < 0.05 compared with dimethyl sulfoxide (DMSO)‐treated control (one‐way ANOVA followed by Dunnett′s test).

Furthermore, we explored drug screening using an FDA‐approved drug library, which contains 765 drugs, to find a modifier of ARSA enzyme activity in fibroblasts. This experiment used GM10199 cells, which showed a slightly higher enzyme activity and missense mutation on both alleles (Table 1). Table 3 shows the cells′ responses to GGA treatment. Figure 5 shows the effects of 765 FDA‐approved drugs at the concentration of 5 *μ*M on the ARSA enzyme activity in GM10199, and relative changes were compared with DMSO‐treated control. Among the 765 FDA‐approved drugs tested, two exhibited an absorbance above 1.2‐fold, higher than the DMSO‐treated control (Figure 5; Drugs A and B). Drug A is fluvoxamine maleate (N06AB08), whereas Drug B is regadenoson (C01EB21). Further, the dose‐dependent effects of fluvoxamine maleate and regadenoson on ARSA enzyme activity were also examined in GM10199 (Figure 6). Fluvoxamine maleate significantly increased enzyme activity at 1 *μ*M but did not change it further up to 50 *μ*M, and it induced cell death at 200 *μ*M. Regadenoson increased enzyme activity at 1 *μ*M but did not change it further up to 200 *μ*M. Overall, the effects of both drugs on enzyme activity were modest, and the drugs did not raise ARSA activity in GM10199 to the level observed in fibroblasts from healthy subjects.

**Figure 5:**
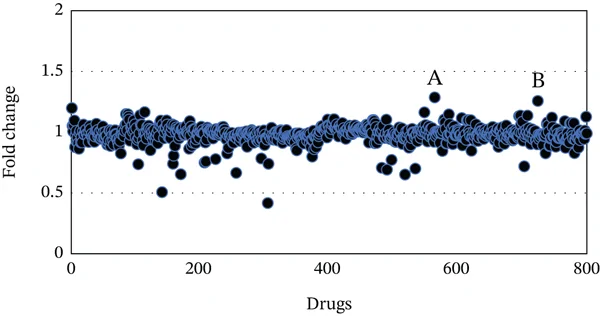
Relative enzyme activity of ARSA in MLD‐derived fibroblasts (GM10199, Passage 13) treated with 765 FDA‐approved drugs. The relative enzyme activity (black circles) in comparison with the dimethyl sulfoxide‐treated control was calculated.

**Figure 6:**
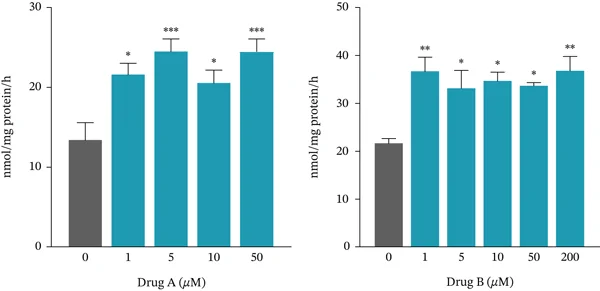
Effects of Drug A (fluvoxamine maleate) and Drug B (regadenoson) on ARSA of MLD‐derived fibroblasts. Cells were treated with either Drug A (fluvoxamine maleate) or Drug B (regadenoson) for 48 h, and ARSA enzyme activity was analyzed in the cells. Mean ± SEM (*n* = 4).  ^∗^ *p* < 0.05,  ^∗∗^ *p* < 0.01, and  ^∗∗∗^ *p* < 0.001 compared with the 0 μM control (one‐way ANOVA followed by Dunnett′s test).

## 4. Discussion

MLD is a progressive demyelinating disease; therefore, appropriate newborn screening methods must be developed, and early‐stage treatment with gene therapy and drug treatment will enhance the patient′s QOL. Fibroblasts derived from MLD patients exhibit reduced ARSA enzyme activity and sulfatide accumulation; therefore, we used these cells to evaluate drug effects and conduct screening. First, our results showed that treatment with GGA, a HSP70 inducer, increased ARSA enzyme activity in the fibroblasts derived from a patient with late‐onset MLD. GGA treatment also reduced mutant ARSA protein levels in the cells, which may reflect enhanced clearance of misfolded/aggregated protein species. Despite this reduction in total protein abundance, the specific enzymatic activity was slightly increased, suggesting that GGA treatment may improve the folding efficiency of the remaining ARSA protein, thereby raising its specific activity. Further studies, including assessment of protein solubility and maturation status (e.g., Endo H sensitivity), are warranted to elucidate the underlying structural alterations and clearance pathways involved.

Next, we searched for chemical chaperone drugs targeting the ARSA enzyme using the fibroblasts. Among the 765 FDA‐approved drugs screened, two (fluvoxamine maleate and regadenoson) increased ARSA enzyme activity by over 1.2‐fold in MLD fibroblasts treated at 5 *μ*M concentration for 48 h compared with DMSO‐treated control. However, the drugs did not exhibit dose‐dependent effects, nor did they restore enzyme activity to levels comparable with those in fibroblasts derived from healthy subjects. Further in vitro and in vivo studies should be conducted to test whether these drugs are effective in alleviating clinical symptoms and slowing the disease progression. To improve drug screening, we need to develop more representative disease models. Drug testing using multiple or prolonged treatments at different concentrations is necessary to understand the interaction of such drugs with the ARSA enzyme in fibroblasts. Conducting drug screenings using the candidate drugs according to in silico enzyme–drug binding assays and the physical properties of mutated ARSA proteins [20–22] would be effective, given that over 200 mutations of ARSA have been reported [23, 24]. Although our focus was on drugs that enhance ARSA enzyme activity, compounds that either inhibit sulfatide synthesis or degrade sulfatide may also represent promising therapeutic strategies for MLD [25, 26]. In particular, iPSC‐derived oligodendrocytes could offer a more relevant cellular context than MLD‐derived fibroblasts [27].

N‐C16:0‐sulfatides accumulated in MLD‐derived fibroblasts compared with those in fibroblasts derived from healthy subjects. Sulfatides are lipids that constitute the myelin sheath; when they accumulate, neuronal defect symptoms, including movement disorders and behavioral problems, may develop in patients with MLD [12, 27]. In addition to the central nervous system, sulfatides also accumulate in various peripheral organs in individuals with MLD. In this study, we confirmed the accumulation of N‐C16:0‐sulfatides in MLD‐derived fibroblasts (Figure 3) and in plasma samples from MLD patients (data not shown). Atidarsagene autotemcel has shown clinical benefit in patients with early‐onset MLD treated in presymptomatic and early‐symptomatic stages of the disease [7], and the gene therapy has since been approved in Europe and the United States for the treatment of MLD patients. Because early intervention is critical for treatment efficacy, newborn screening for MLD using dried blood spots (DBS) is being developed worldwide. Screening is carried out by measurement of the elevation of sulfatides, such as N‐C16:0‐ and 16:1‐OH‐sulfatides, in DBS, followed by measurement of ARSA enzyme activity in the same DBS. We have been developing an LC‐MS/MS method to detect sulfatides in DBS, and this method has successfully identified MLD patients (unpublished data). However, in the current experiment, we did not examine whether a reduction in N‐C16:0‐sulfatide levels occurred following 48‐h treatment with GGA, fluvoxamine maleate, or regadenoson in MLD fibroblasts. As far as we know, clinical monitoring in gene therapy for MLD has largely relied on restoration of ARSA enzymatic activity in blood and CSF, rather than sulfatide measurements [7], whereas in animal studies, sulfatide levels in brain tissue and CSF have been used to confirm therapeutic efficacy [28, 29]. Given that sulfatide accumulation directly underlies disease pathology, incorporating sulfatide measurement into the monitoring of gene therapy may provide a more direct assessment of therapeutic efficacy in patients, and warrants further investigation in future clinical studies.

## Author Contributions

Conceptualization: Y.E. Funding acquisition: Y.E. Project administration: Y.E. and T.I. Investigation: T.M., M.I., and C.W. Data curation: T.M. and M.I. Visualization: T.M. and M.I. Writing—original draft: Y.E. Writing—review and editing: T.M., M.I., C.W., T.I., and Y.E. Y.E. has primary responsibility for the final manuscript.

## Funding

This study was supported by Sanofi (10.13039/100004339; SGZ201912859) and Actelion Pharmaceuticals (10.13039/100005646).

## Disclosure

All authors approved the final version of the manuscript for submission.

## Conflicts of Interest

Yoshikatsu Eto has received research grants from Sanofi Co. and Actelion Pharmaceuticals.

## Data Availability

The data that support the findings of this study are available from the corresponding author upon reasonable request.
